# Modelling the effect of malaria endemicity on spatial variations in childhood fever, diarrhoea and pneumonia in Malawi

**DOI:** 10.1186/1476-072X-6-33

**Published:** 2007-07-25

**Authors:** Lawrence N Kazembe, Adamson S Muula, Christopher C Appleton, Immo Kleinschmidt

**Affiliations:** 1 Applied Statistics and Epidemiology Research Unit, Mathematical Sciences Department, Chancellor College, University of Malawi, Zomba, Malawi; 2 Malaria Research Programme, Medical Research Council, Durban, South Africa; 3 Department of Community Health, College of Medicine, University of Malawi, Blantyre, Malawi; 4 School of Biological and Conservation Sciences, University of KwaZulu-Natal, Durban, South Africa; 5 Infectious Diseases Epidemiology Unit, Department of Epidemiology and Population Health, London School of Hygiene and Tropical Medicine, London, UK

## Abstract

**Background:**

Co-morbidity with conditions such as fever, diarrhoea and pneumonia is a common phenomenon in tropical Africa. However, little is known about geographical overlaps in these illnesses. Spatial modelling may improve our understanding of the epidemiology of the diseases for efficient and cost-effective control.

**Methods:**

This study assessed subdistrict-specific spatial associations of the three conditions (fever, diarrhoea and pneumonia) in relation to malaria endemicity. We used data from the 2000 Malawi demographic and health survey which captured the history of childhood morbidities 2 weeks prior to the survey date. The disease status of each child in each area was the outcome of interest and was modelled using a trivariate logistic regression model, and incorporated random effects to measure spatial correlation.

**Results:**

The risk of fever was positively associated with high and medium malaria endemicity levels relative to low endemicity level, while for diarrhoea and pneumonia we observed marginal positive association at high endemicity level relative to low endemicity level, controlling for confounding covariates and heterogeneity. A positive spatial correlation was found between fever and diarrhoea (*r *= 0.29); while weak associations were estimated between fever and pneumonia (*r *= 0.01); and between diarrhoea and pneumonia (*r *= 0.05). The proportion of structured spatial variation compared to unstructured variation was 0.67 (95% credible interval (CI): 0.31–0.91) for fever, 0.67 (95 % CI: 0.27–0.93) for diarrhoea, and 0.87 (95% CI: 0.62–0.96) for pneumonia.

**Conclusion:**

The analysis suggests some similarities in subdistrict-specific spatial variation of childhood morbidities of fever, diarrhoea and pneumonia, and might be a result of shared and overlapping risk factors, one of which is malaria endemicity.

## Background

Children in sub-Saharan Africa experience a disproportionately large burden of morbidity and mortality. About 180 deaths per 1000 live births occur in the region [1], mostly from a relatively small number of infectious diseases [2]. Often these illnesses occur simultaneously because of common risk factors and probably due to overlaps between multiple risk factors, or because one disorder creates an increased risk for the other [3].

One of the common childhood co-morbidities is of diarrhoea, malaria, HIV and acute respiratory illnesses such as pneumonia [4,5]. Each disease has its own aetiology and environmental or behavioural covariates synergistically expedite severe disease or death [6,7]. In settings where malaria risk is perennial, the many febrile conditions children experience have been attributed to malaria risk. For example, most feverish conditions reported are a direct cause of malaria infections [8]. At the same time, immuno-suppression as a consequence of malaria infection tend to increase the risk of other illnesses including diarrhoea and pneumonia [2,5-7]. In Malawi, malaria remains highly endemic because of lack of sustainable control programmes, with an estimated one million children between ages of between 1 and 10 years living in medium to high risk areas [9].

The HIV epidemic in sub-Saharan Africa exacerbates the risk of morbidity and mortality to which children are exposed. The HIV prevalence among adults aged 15–49 years in Malawi is estimated at 14.1% (range: 6.9–21.4%), 91,000 children (range: 28,000–190,000) are living with HIV [10]. HIV infection doubles the risk of malaria parasitemia and clinical malaria [4]. Symptoms of HIV include fever and diarrhoea, and pneumonia is a common opportunistic infection associated with HIV. The World Health Organisation (WHO) has proposed an Integrated Management of Childhood Illnesses programme that takes account of the prevalence of HIV. However, lack of HIV prevalence data among children means that the effect of this infection cannot be directly quantified.

This study applied a spatial model to investigate the effect of malaria endemicity on childhood co-morbidity of fever, diarrhoea and pneumonia. This relationship was investigated using the 2000 Malawi Demographic and Health Survey (DHS) databases and data from the Mapping Malaria Risk in Africa (MARA). The DHS survey contains data on childhood health, and included questions on childhood fever, diarrhoea and pneumonia. Malaria risk was based on prevalence predicted at precise DHS survey sampling locations, by applying a geostatistical model developed and described in Kazembe *et al *[9].

Since high malaria risk is likely to affect other diseases, it was important to examine its effect on the spatial patterns of childhood fever, diarrhoea and pneumonia. Moreover, as fever, diarrhoea and pneumonia share risk factors [3,11], spatial association across areas would be expected. The analysis of geographical variation in these morbidities is important for identifying areas of excessive inequalities in health outcomes. Explaining variation of more than one disease can give clues about common risk factors. An appropriate health delivery response would be an integrated management strategy of the diseases, including initiating unified home and community management of malaria, pneumonia and diarrhoea [12,13].

Spatial models have been applied in previous studies [11,14], in which district-specific geographical variation in childhood fever and diarrhoea were analysed, fitting separate models for each disease. In this study a multivariate spatial model [15], was applied to analyse more than one disease simultaneously. The advantage of this is that one can quantify the correlation between relative risks for each disease as well as enable disease-specific residuals to be mapped, while at the same time, examining the influence of covariates on each disease. Specifically, the objectives of this paper were to 1) describe the spatial variation of malarial fever, diarrhoea and pneumonia at subdistrict level in Malawi, 2) assess the influence of malaria endemicity, adjusting for confounding individual-level covariates; 3) estimate the correlation between diseases at subdistrict level.

## Methods

### Data

The analysis used self-reported data from the 2000 Malawi Demographic and Health Survey (MDHS) [16]. The MDHS employed a multi-stage sampling design, stratified by region and urban/rural status, with sampling probability proportional to the population of selected enumeration areas (EAs). A total of 13,220 women aged 15–49 years, sampled from 560 EAs, were interviewed on various health issues. Women were asked about children under 5 in their households who had recent episodes of fever, diarrhoea and pneumonia. Questions used to determine recent episodes of fever, diarrhoea and pneumonia respectively were: "Does the child have fever now/Did the child have fever during the last 2 weeks", "Did the child have diarrhoea in the last 2 weeks", "Did the child have an illness with coughing, did he/she breathe faster than usual with short, fast breaths".

The self-reported sickness status (0/1) of each child for each disease was the outcome of interest. The data set contained 4,778 cases where responses to all three illnesses were available. The following individual covariates were included in the analysis: (1) age of the child; (2) ownership of bednets (1 = yes, 0 = no); (3) received vitamin A within 6 months prior to the survey date (1 = yes, 0 = no); (4) weight-for-age as a general indicator of nutritional status, measured as Z-scores; (5) type of place of residence (1 = rural, 0 = urban); (6) crowding indicator based on whether household size exceeded 6 members (1 = yes, 0 = no).

**Figure 1:**
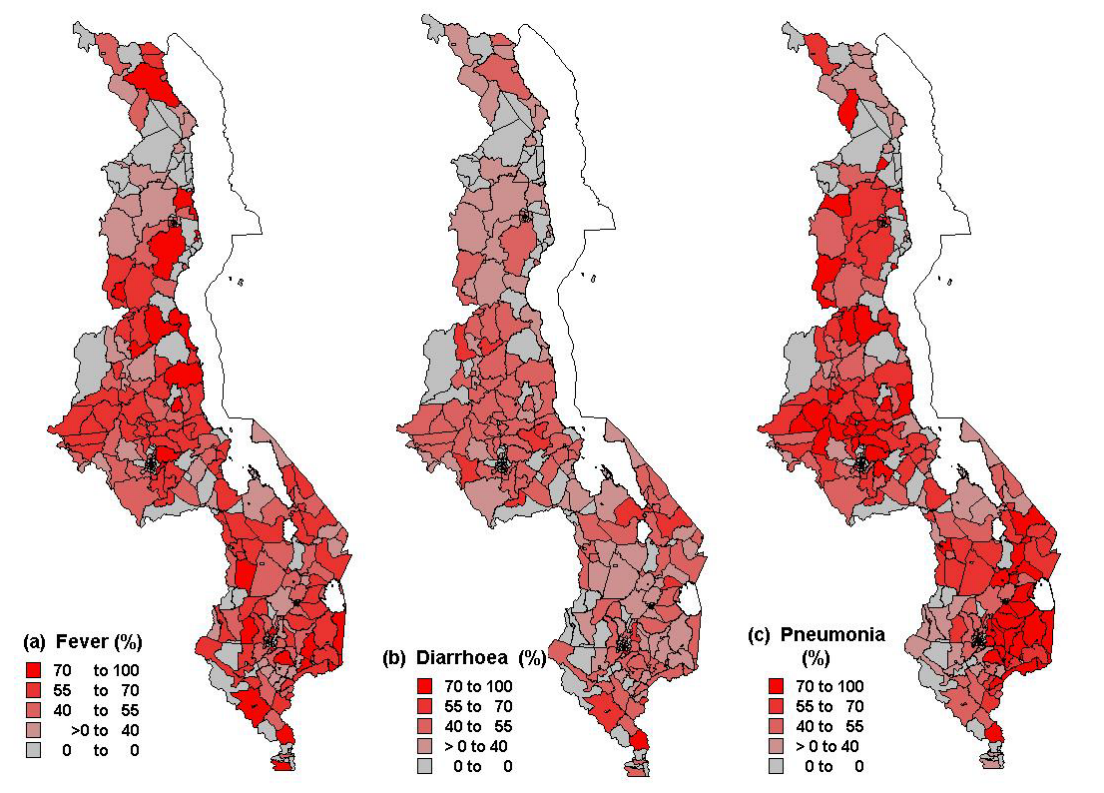
Relative frequency of: (a) childhood fever (b) childhood diarrhoea (c) childhood pneumonia by sub-districts.

At community level (i.e. at all 560 EAs), malaria endemicity (measured using prevalence of infection) was included, assuming that each child at each site was equally exposed to the underlying risk. Malaria prevalence was predicted for MDHS precise sampling locations using a geostatistical model developed and described elsewhere [9]. The malaria data were from children of age ≤ 10 years at 73 different sites, across the country, where malariometric surveys were conducted. Predicted prevalence values were categorized into three groups: low (0–35%: reference category), medium (36–60%) and high endemicity (61–100%). Table 1 gives summary statistics of all variables used in the analysis. The map (Figure 1), shows the observed percentages of childhood fever, diarrhoea and pneumonia across sub-districts.

**Table 1:** Areal and individual characteristics for children who had fever, diarrhoea or pneumonia during the two weeks preceding the survey, Malawi DHS 2000.

Variable		Fever^†^	Diarrhoea	Pneumonia	*n *= 4778
*Areal characteristics*					
Mean number (*n*) sick per sub-district (range)		11 (1–60)	5 (1–26)	12 (1–63)	20 (1–88)
^‡^Proportion sick per sub-district (range), %		50 (0–100)	21 (0–75)	53 (0–100)	
*Individual characteristics*					
Age of child	0–5 months	47.2	16	57.1	616
	6–11 months	66.5	39.7	59.7	668
	12–23 months	62.1	36.7	58.5	1068
	24–35 months	54.5	16.8	54.9	930
	36–59 months	42.6	9.5	52.3	1468
Malaria prevalence	Low (0–35%)	48.3	19.4	55.5	1005
	Medium (36–60%)	53.5	21.9	54.3	2940
	High (>60%)	57.4	25.6	61.5	933
Bednet ownership	No	53.9	22.7	57.3	3998
	Yes	49.5	19.0	49.4	780
Vitamin A supplement	No	48.7	20.1	54.0	1656
	Yes	55.5	23.2	57.0	3122
Residence	Urban	47.2	18.6	46.5	779
	Rural	54.3	22.8	57.8	3999
Crowded household	No	53.7	21.6	56.3	1480
	Yes	52.9	22.3	55.8	3298
Weight-for-Age (Z scores)	Mean	-0.06	-0.19	-0.008	
	St.dev	0.98	0.91	1.01	

^‡^Proportion sick during preceding 2 weeks

^†^Numbers under each disease column are percentage unless stated otherwise

### Statistical modelling

Assume *y*_*ijk *_is the status (0/1) of disease *k*, *k *= 1 (fever), 2 (diarrhoea), 3 (pneumonia) for child *j *in subdistrict *i*, *i *= 1, . . .,364. Suppose that the observed outcomes arise from a trivariate Bernoulli distribution, with *p*_*ijk *_as the probability of disease *k *occurring in child *j *in area *i*, then the outcome is modelled using a logistic regression model with predictor given by,

η=(log⁡it(pij1)log⁡it(pij2)log⁡it(pij3))=(α1α2α3)+XijkT(β1β2β3)+(si1si2si3)+(ui1ui2ui3)
 MathType@MTEF@5@5@+=feaafiart1ev1aaatCvAUfKttLearuWrP9MDH5MBPbIqV92AaeXatLxBI9gBaebbnrfifHhDYfgasaacH8akY=wiFfYdH8Gipec8Eeeu0xXdbba9frFj0=OqFfea0dXdd9vqai=hGuQ8kuc9pgc9s8qqaq=dirpe0xb9q8qiLsFr0=vr0=vr0dc8meaabaqaciaacaGaaeqabaqabeGadaaakeaaiiWacqWF3oaAcqGH9aqpdaqadaqaauaabeqadeaaaeaacyGGSbaBcqGGVbWBcqGGNbWzcqqGPbqAcqqG0baDcqGGOaakcqWGWbaCdaWgaaWcbaGaemyAaKMaemOAaOMaeGymaedabeaakiabcMcaPaqaaiGbcYgaSjabc+gaVjabcEgaNjabbMgaPjabbsha0jabcIcaOiabdchaWnaaBaaaleaacqWGPbqAcqWGQbGAcqaIYaGmaeqaaOGaeiykaKcabaGagiiBaWMaei4Ba8Maei4zaCMaeeyAaKMaeeiDaqNaeiikaGIaemiCaa3aaSbaaSqaaiabdMgaPjabdQgaQjabiodaZaqabaGccqGGPaqkaaaacaGLOaGaayzkaaGaeyypa0ZaaeWaaeaafaqabeWabaaabaacciGae4xSde2aaSbaaSqaaiabigdaXaqabaaakeaacqGFXoqydaWgaaWcbaGaeGOmaidabeaaaOqaaiab+f7aHnaaBaaaleaacqaIZaWmaeqaaaaaaOGaayjkaiaawMcaaiabgUcaRGqadiab9HfaynaaDaaaleaacqWGPbqAcqWGQbGAcqWGRbWAaeaacqWGubavaaGcdaqadaqaauaabeqadeaaaeaacqWFYoGydaWgaaWcbaGaeGymaedabeaaaOqaaiab=j7aInaaBaaaleaacqaIYaGmaeqaaaGcbaGae8NSdi2aaSbaaSqaaiabiodaZaqabaaaaaGccaGLOaGaayzkaaGaey4kaSYaaeWaaeaafaqabeWabaaabaGaem4Cam3aaSbaaSqaaiabdMgaPjabigdaXaqabaaakeaacqWGZbWCdaWgaaWcbaGaemyAaKMaeGOmaidabeaaaOqaaiabdohaZnaaBaaaleaacqWGPbqAcqaIZaWmaeqaaaaaaOGaayjkaiaawMcaaiabgUcaRmaabmaabaqbaeqabmqaaaqaaiabdwha1naaBaaaleaacqWGPbqAcqaIXaqmaeqaaaGcbaGaemyDau3aaSbaaSqaaiabdMgaPjabikdaYaqabaaakeaacqWG1bqDdaWgaaWcbaGaemyAaKMaeG4mamdabeaaaaaakiaawIcacaGLPaaaaaa@93BE@

where *α*_*k *_is the intercept for disease *k*, the terms ***β ***= (***β***_1_, ***β***_2_, ***β***_3_)^*T *^are vectors of regression parameters corresponding to the set of covariates (***X***_*ijk*_) (Table 1). The components *u*_*ik *_and *s*_*ik *_are the unstructured heterogeneity and spatially structured variation terms, respectively, at subdistrict level. Because of dependent binary outcomes, the random effects are correlated and are modelled using multivariate normal distributions as explained below.

Model estimation was carried out using the Bayesian approach and the following prior distributions were specified for all parameters of the model (1). Without empirical evidence about the magnitude and direction of covariate effects, non-informative priors were assigned to the regression coefficients. For the intercept, diffuse priors were assumed, that is, *p*(*α*_*k*_) ∝ 1, while for the other fixed effects, ***β***, highly dispersed normal distribution priors were chosen, that is, *p*(***β***) ~ *N*(0, 10000). The unstructured spatial effects *u*_*ik *_were assumed to follow a multivariate normal distribution, i.e., (*u*_*i*1_, *u*_*i*2_, *u*_*i*3_)^*T *^~ *MVN*(0, Ω), with covariance matrix Ω. The spatial structured effects *s*_*ik *_were assigned a multivariate conditional autoregressive (MCAR) prior, i.e.,(*s*_*i*1_, *s*_*i*2_, *s*_*i*3_)^*T *^~ *MCAR*(1, Σ), again Σ is a covariance matrix [15].

The covariance matrices of the spatiall effects have their diagonal elements equal to the variances and the off-diagonals are correlation components between the diseases. Thus, for example Σ_11_, Σ_22_, Σ_33 _are variance components corresponding to fever, diarrhoea and pneumonia respectively, while Σ_12_, Σ_13_, Σ_23 _are correlation components between fever and diarrhoea, fever and pneumonia and diarrhoea and pneumonia respectively. Correspondingly, *r*_12 _= r12=Σ12Σ11Σ22
 MathType@MTEF@5@5@+=feaafiart1ev1aaatCvAUfKttLearuWrP9MDH5MBPbIqV92AaeXatLxBI9gBaebbnrfifHhDYfgasaacH8akY=wiFfYdH8Gipec8Eeeu0xXdbba9frFj0=OqFfea0dXdd9vqai=hGuQ8kuc9pgc9s8qqaq=dirpe0xb9q8qiLsFr0=vr0=vr0dc8meaabaqaciaacaGaaeqabaqabeGadaaakeaacqWGYbGCdaWgaaWcbaGaeGymaeJaeGOmaidabeaakiabg2da9maalaaabaGaeu4Odm1aaSbaaSqaaiabigdaXiabikdaYaqabaaakeaadaGcaaqaaiabfo6atnaaBaaaleaacqaIXaqmcqaIXaqmaeqaaOGaeu4Odm1aaSbaaSqaaiabikdaYiabikdaYaqabaaabeaaaaaaaa@3C21@, for example, gives a measure of spatial correlation between fever and diarrhoea. The variance components, at a further stage, were assigned inverse Wishart priors, i.e., Ω ~ *IW *(*q*, *Q*), Σ ~ *IW *(*r*, *R*) where *q*,*r *are scalars, while *Q*, *R *are symmetric and positive definitive matrices. The hyperpriors were assigned *q *= *r *= 3, *Q *= *R *= 0.01*I *where *I *is an identity matrix.

Model fitting used Markov Chain Monte Carlo simulation techniques to draw samples from the posterior distribution and was implemented in WinBugs 1.4 [17]. Three parallel chains were run to help assess convergence, starting from different initial dispersed values for all the parameters. Each model quantity was monitored from the first iteration. Convergence was evaluated by inspecting trace and autocorrelation plots of samples for each chain, as well as through numerical summaries such as the R
 MathType@MTEF@5@5@+=feaafiart1ev1aaatCvAUfKttLearuWrP9MDH5MBPbIqV92AaeXatLxBI9gBaebbnrfifHhDYfgasaacH8akY=wiFfYdH8Gipec8Eeeu0xXdbba9frFj0=OqFfea0dXdd9vqai=hGuQ8kuc9pgc9s8qqaq=dirpe0xb9q8qiLsFr0=vr0=vr0dc8meaabaqaciaacaGaaeqabaqabeGadaaakeaadaGcaaqaaiabdkfasbWcbeaaaaa@2DF4@ diagnostic statistic of Brooks et al. [18]. After 5,000 iterations, all parameters showed signs of convergence in the trace plots. The values of R
 MathType@MTEF@5@5@+=feaafiart1ev1aaatCvAUfKttLearuWrP9MDH5MBPbIqV92AaeXatLxBI9gBaebbnrfifHhDYfgasaacH8akY=wiFfYdH8Gipec8Eeeu0xXdbba9frFj0=OqFfea0dXdd9vqai=hGuQ8kuc9pgc9s8qqaq=dirpe0xb9q8qiLsFr0=vr0=vr0dc8meaabaqaciaacaGaaeqabaqabeGadaaakeaadaGcaaqaaiabdkfasbWcbeaaaaa@2DF4@ also quickly approached 1 and were all below the value of 1.12, which indicated convergence of both the pooled and within interval widths to stability. The first 10,000 pre-convergence samples were then discarded as "burn-in" and each chain was run for a further 20,000 iterations for parameter estimation, with Monte Carlo errors <5% of the posterior standard deviation.

Because of the known concerns about the Wishart prior's possible informativity, a sensitivity analysis was carried out. Three specifications, i.e., *Q *= *R *= 0.005, 0.01,0.05 with *q *= *r *= 3, were carried out and the MCMC simulations were re-run for each choice. Using these priors gave satisfactory results, and although the prior can give problems on convergence, we did not have any problem on convergence or mixing of the chains in this application. The estimates of fixed and random effects, obtained from the posterior distributions, were similar indicating that the results were not sensitive to changes in prior distributions.

## Results

Table 1 gives summary statistics for the areal and individual characteristics. The mean number of children reported sick per subdistrict was 11 (range: 1–60) for fever, 5 (range: 1–26) for diarrhoea, and 12 (range: 1–63) for pneumonia in the sample of *n *= 4,778 children. The corresponding proportions were 50% (range: 0–100), 21% (range: 0–75) and 53% (range: 0–100) for fever, diarrhoea and pneumonia respectively. Very young infants (age 0 to 5 months) and older children (36 to 59 months) were less likely to be sick compared to the other age groups across all the three diseases. The proportions sick were more at higher malaria endemicity levels for all illnesses. Diarrhoea occurred mostly in underweight children. Rural children were disproportionately more sick than their urban counterparts. Those with bednets were less sick than those without.

### Fixed effects of childhood morbidities

Table 2 provides estimates for the fixed effects. The risk of fever was found to be associated with malaria endemicity at both medium (Odds ratio (OR) = 1.26, 95% Credible Interval (CI): 1.03–1.61) and high endemic levels (OR = 1.48, 95% CI: 1.08–2.06) relative to low levels. The association with diarrhoea was marginal at high endemicity level (OR = 1.35, 95% CI:0.97–1.88) relative to low endemicity level. At medium level we observed positive association, though, not significant (OR = 1.12, 95% CI: 0.87–1.44). The risk of pneumonia was marginally associated with high malaria endemicity level relative to low endemicity level (OR = 1.47, 95% CI: 0.95–2.08), and similar at medium level relative to low endemicity level (OR = 1.02, 95% CI: 0.82–1.30).

**Table 2:** Fixed effects estimates from the joint spatial model of childhood fever, diarrhoea and pneumonia morbidity in Malawi, 2000.

		Fever		Diarrhoea		Pneumonia	
		OR	95%CI	OR	95%CI	OR	95%CI
*Fixed Effects*							
Malaria prevalence	Low: 0–35%	1.00		1.00		1.00	
	Medium: 36–60%	1.26	(1.03, 1.61)	1.12	(0.87, 1.44)	1.02	(0.82, 1.30)	
	High:>60%	1.48	(1.08, 2.06)	1.35	(0.97, 1.88)	1.47	(0.95, 2.08)
Residence	Rural	1.20	(1.01, 1.45)	1.16	(0.92, 1.46)	1.48	(1.24, 1.76)
	Urban	1.00		1.00		1.00	
Age of child	0–5 months	1.47	(1.20, 1.83)	2.35	(1.76, 3.19)	1.23	(0.98, 1.50)
	6–11 months	2.84	(2.39, 3.47)	6.98	(5.49, 9.02)	1.35	(1.12, 1.64)
	12–23 months	2.24	(1.90, 2.64)	5.72	(4.55, 7.18)	1.33	(1.12, 1.57)
	24–35 months	1.65	(1.40, 1.96)	1.92	(1.50, 2.47)	1.10	(0.92, 1.29)
	36–59 months	1.00		1.00		1.00	
Vitamin A supplement	No	1.00		1.00		1.00	
	Yes	1.21	(1.06, 1.38)	0.96	(0.81, 1.13)	1.12	(0.99, 1.28)
Bednet ownership	No	1.00		1.00		1.00	
	Yes	0.85	(0.72, 1.02)	0.84	(0.67, 1.04)	1.02	(0.86, 1.22)
Crowded household	No	1.00		1.00		1.00	
	Yes	0.95	(0.83, 1.08)	1.04	(0.88, 1.22)	0.98	(0.85, 1.11)
Weight-for-Age		0.90	(0.84, 0.95)	0.77	(0.71, 0.83)	1.01	(0.95, 1.08)

OR-Odds ratio; CI-Credible interval

The risk of childhood fever increased with rural residence relative to urban residence (OR = 1.20, 95% CI: 1.01–1.45). Children aged 0–5, 6–11, 12–23, 24–35 months relative to 36–59 months were at higher risk of fever (Table 2). Those who received Vitamin A relative to those who did not were at increased risk of fever. Net ownership and weight for age were associated with lower risk of fever. Risk factors positively associated with diarrhoea were rural place of residence and all children aged below three years. Lower risk of diarrhoea was associated with bed nets ownership and weight for age (Table 2). Pneumonia was positively associated with children younger than three years of age, rural place of residence, weight for age, and Vitamin A uptake (Table 2).

### Spatial effects of childhood morbidities

The degree of spatial heterogeneity is given by Σ and Ω in Table 3. The spatially structured variation was estimated as Σ_11 _= 0.62 (95% CI: 0.41–1.01) for fever, Σ_22 _= 0.27 (95% CI: 0.16–0.51) for diarrhoea, and Σ_33 _= 0.88 (95% CI: 0.59–1.31) for pneumonia. For the unstructured heterogeneity, we estimated Ω_11 _= 0.43 (95% CI: 0.25–0.94) for fever, Ω_22 _= 0.19 (95% CI: 0.10–0.36) for diarrhoea, and Σ_33 _= 0.34 (95% CI: 0.21–0.59) for pneumonia. The proportion of variation due to the spatially structured variation was estimated as *p*_11 _= 0.67 (95% CI: 0.29–0.91) for fever, *p*_22 _= 0.67 (95% CI: 0.28–0.93) for diarrhoea, and *p*_33 _= 0.87 (95% CI: 0.63–0.96) for pneumonia.

**Table 3:** Random effects estimates from the joint spatial model of childhood fever, diarrhoea and pneumo nia morbidity in Malawi, 2000.

		Fever Post. Mean^‡^ (95% CI)	Diarrhoea Post. Mean (95% CI)	Pneumonia Post. Mean (95% CI)
*Random effects*				
Spatial structured	(Σ_11_, Σ_22_, Σ_33_)	0.62	0.27	0.88
		(0.41, 1.01)	(0.16, 0.51)	(0.59, 1.31)
Spatial unstructured	(Ω_11_, Ω_22_, Ω_33_)	0.43	0.19	0.34
		(0.25, 0.94)	(0.10, 0.36)	(0.21, 0.59)
Proportion of structured				
spatial variation	(*p*_11_, *p*_22_, *p*_33_)	0.67	0.67	0.87
		(0.29, 0.91)	(0.28, 0.93)	(0.63, 0.96)
*Correlation structured*				
Fever	r12=Σ12Σ11Σ22,r13=Σ13Σ11Σ33 MathType@MTEF@5@5@+=feaafiart1ev1aaatCvAUfKttLearuWrP9MDH5MBPbIqV92AaeXatLxBI9gBaebbnrfifHhDYfgasaacH8akY=wiFfYdH8Gipec8Eeeu0xXdbba9frFj0=OqFfea0dXdd9vqai=hGuQ8kuc9pgc9s8qqaq=dirpe0xb9q8qiLsFr0=vr0=vr0dc8meaabaqaciaacaGaaeqabaqabeGadaaakeaacqWGYbGCdaWgaaWcbaGaeGymaeJaeGOmaidabeaakiabg2da9maalaaabaGaeu4Odm1aaSbaaSqaaiabigdaXiabikdaYaqabaaakeaadaGcaaqaaiabfo6atnaaBaaaleaacqaIXaqmcqaIXaqmaeqaaOGaeu4Odm1aaSbaaSqaaiabikdaYiabikdaYaqabaaabeaaaaGccqGGSaalcqWGYbGCdaWgaaWcbaGaeGymaeJaeG4mamdabeaakiabg2da9maalaaabaGaeu4Odm1aaSbaaSqaaiabigdaXiabiodaZaqabaaakeaadaGcaaqaaiabfo6atnaaBaaaleaacqaIXaqmcqaIXaqmaeqaaOGaeu4Odm1aaSbaaSqaaiabiodaZiabiodaZaqabaaabeaaaaaaaa@4C88@	1.00	0.18	0.02
			(-0.48, 0.72)	(-0.56, 0.56)
Diarrhoea	r23=Σ23Σ22Σ33 MathType@MTEF@5@5@+=feaafiart1ev1aaatCvAUfKttLearuWrP9MDH5MBPbIqV92AaeXatLxBI9gBaebbnrfifHhDYfgasaacH8akY=wiFfYdH8Gipec8Eeeu0xXdbba9frFj0=OqFfea0dXdd9vqai=hGuQ8kuc9pgc9s8qqaq=dirpe0xb9q8qiLsFr0=vr0=vr0dc8meaabaqaciaacaGaaeqabaqabeGadaaakeaacqWGYbGCdaWgaaWcbaGaeGOmaiJaeG4mamdabeaakiabg2da9maalaaabaGaeu4Odm1aaSbaaSqaaiabikdaYiabiodaZaqabaaakeaadaGcaaqaaiabfo6atnaaBaaaleaacqaIYaGmcqaIYaGmaeqaaOGaeu4Odm1aaSbaaSqaaiabiodaZiabiodaZaqabaaabeaaaaaaaa@3C31@		1.00	0.04
				(-0.56, 0.63)
Pneumonia				1.00
*Correlation unstructured*				
Fever	q12=Ω12Ω11Ω22,q13=Ω13Ω11Ω33 MathType@MTEF@5@5@+=feaafiart1ev1aaatCvAUfKttLearuWrP9MDH5MBPbIqV92AaeXatLxBI9gBaebbnrfifHhDYfgasaacH8akY=wiFfYdH8Gipec8Eeeu0xXdbba9frFj0=OqFfea0dXdd9vqai=hGuQ8kuc9pgc9s8qqaq=dirpe0xb9q8qiLsFr0=vr0=vr0dc8meaabaqaciaacaGaaeqabaqabeGadaaakeaacqWGXbqCdaWgaaWcbaGaeGymaeJaeGOmaidabeaakiabg2da9maalaaabaGaeuyQdC1aaSbaaSqaaiabigdaXiabikdaYaqabaaakeaadaGcaaqaaiabfM6axnaaBaaaleaacqaIXaqmcqaIXaqmaeqaaOGaeuyQdC1aaSbaaSqaaiabikdaYiabikdaYaqabaaabeaaaaGccqGGSaalcqWGXbqCdaWgaaWcbaGaeGymaeJaeG4mamdabeaakiabg2da9maalaaabaGaeuyQdC1aaSbaaSqaaiabigdaXiabiodaZaqabaaakeaadaGcaaqaaiabfM6axnaaBaaaleaacqaIXaqmcqaIXaqmaeqaaOGaeuyQdC1aaSbaaSqaaiabiodaZiabiodaZaqabaaabeaaaaaaaa@4CC0@	1.00	0.47	-0.01
			(-0.32, 0.87)	(-0.64, 0.68)
Diarrhoea	q23=Ω23Ω2Ω33 MathType@MTEF@5@5@+=feaafiart1ev1aaatCvAUfKttLearuWrP9MDH5MBPbIqV92AaeXatLxBI9gBaebbnrfifHhDYfgasaacH8akY=wiFfYdH8Gipec8Eeeu0xXdbba9frFj0=OqFfea0dXdd9vqai=hGuQ8kuc9pgc9s8qqaq=dirpe0xb9q8qiLsFr0=vr0=vr0dc8meaabaqaciaacaGaaeqabaqabeGadaaakeaacqWGXbqCdaWgaaWcbaGaeGOmaiJaeG4mamdabeaakiabg2da9maalaaabaGaeuyQdC1aaSbaaSqaaiabikdaYiabiodaZaqabaaakeaadaGcaaqaaiabfM6axnaaBaaaleaacqaIYaGmaeqaaOGaeuyQdC1aaSbaaSqaaiabiodaZiabiodaZaqabaaabeaaaaaaaa@3B5B@		1.00	0.09
				(-0.62, 0.73)
Pneumonia				1.00
*Total correlation *(*q *+ *r*)				
Fever		1.00	0.29	0.01
			(-0.26, 0.69)	(-0.45, 0.46)
Diarrhoea			1.00	0.05
				(-0.43, 0.51)
Pneumonia				1.00

^‡^Pos. Mean = posterior mean; CI = Credible interval

The correlations between diseases at subdistrict level are also presented in Table 3. The correlations associated with spatially structured variation, Σ, were *r*_12 _= 0.18 (95% CI: -0.48–0.72) for fever and diarrhoea; *r*_13 _= 0.02 (95% CI: -0.56–0.56) for fever and pneumonia; and *q*_23 _= 0.04 (95% CI: -0.56–0.63) for diarrhoea and pneumonia. For the unstructured heterogeneity, the correlations were *q*_12 _= 0.47 (95% CI: -0.32–0.87) for fever and diarrhoea, *q*_13 _= -0.01 (95% CI: -0.64–0.68) for fever and pneumonia, while *q*_23 _= 0.09 (95% CI: -0.62–0.73) for diarrhoea and pneumonia. The total spatial correlations (*q *+ *r*) were 0.29 (95% CI: -0.26–0.69), 0.01 (95% interval: -0.45–0.46), and 0.05 (95% CI: -0.43–0.51) for fever and diarrhoea, fever and pneumonia; and diarrhoea and pneumonia respectively.

**Figure 2:**
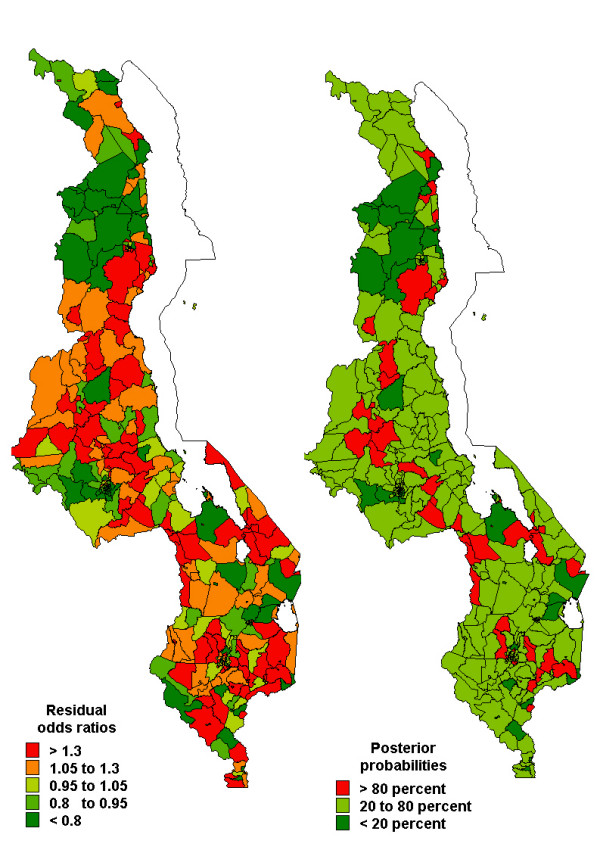
Spatial residual effects of childhood fever, MDHS 2000. Left map shows odds ratios (OR) per subdistrict compared to the overall mean. Right map shows the corresponding posterior probabilities for OR>1: <20 percent deep green, 20–80 percent light green, >80 percent red.

**Figure 3:**
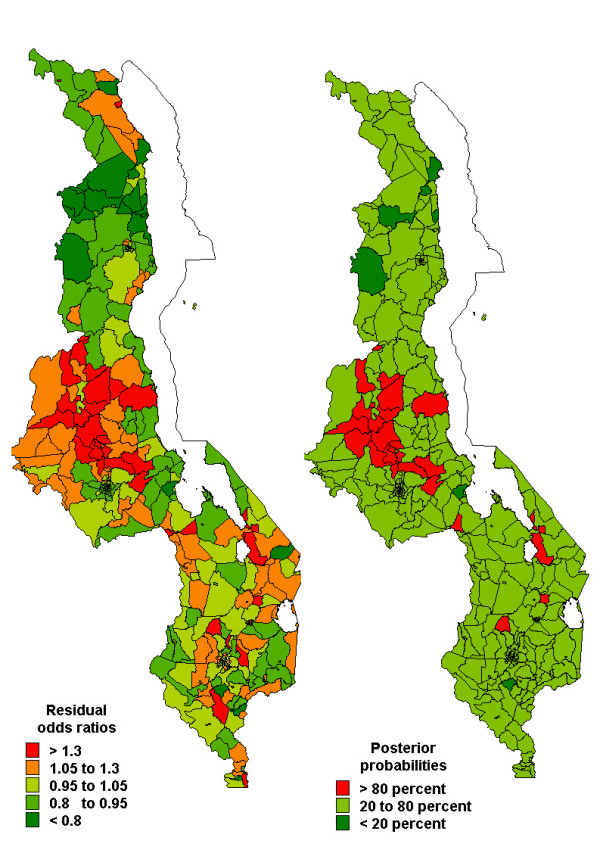
Spatial residual effects of childhood diarrhoea, MDHS 2000. Left map shows odds ratios (OR) per subdistrict compared to the overall mean. Right map shows the corresponding posterior probabilities for OR>1: <20 percent deep green, 20–80 percent light green, >80 percent red.

**Figure 4:**
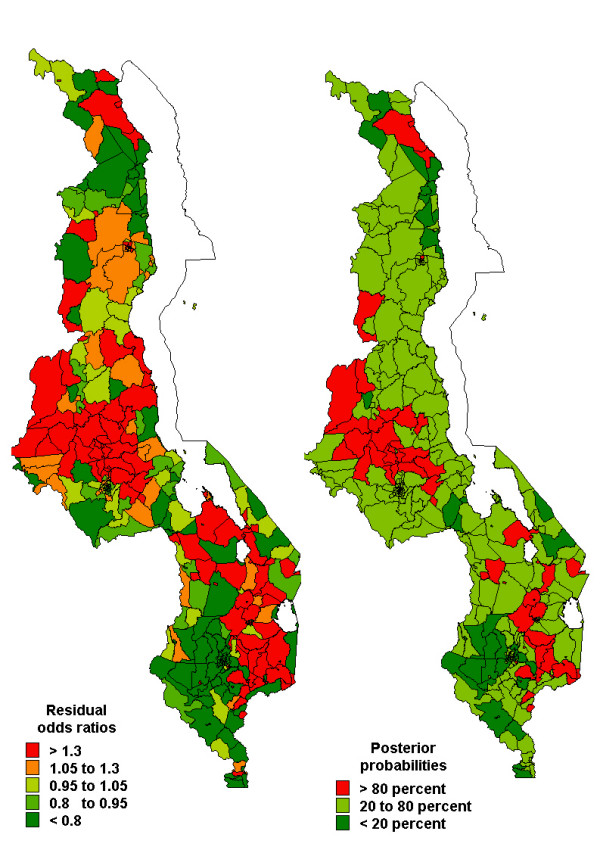
Spatial residual effects of childhood pneumonia, MDHS 2000. Left map shows odds ratios (OR) per subdistrict compared to the overall mean. Right map shows the corresponding posterior probabilities for OR>1: <20 percent deep green, 20–80 percent light green, >80 percent red.

Figures 2, 3, 4 show the spatial residual effects for fever, diarrhoea and pneumonia. The accompanying right map highlights areas where OR>1 is above 80% or below 20%, in other words, the map shows areas where spatial clusters of risk occur based on Richardson's criterion [19]. This criterion recommends that probabilities over 80% be deemed positively significant, those below 20% be judged negatively significant, while those between 20 and 80% be considered not significant. In Figure 2, increased risk of fever appeared in the central and southern region, while decreased risk was mainly in the northern region. Areas of high probability (red colour) were concentrated in the central region, while those of low probability (green colour) were in urban areas and the northern region. However, in most areas the probability was between 0.2–0.8 (not significant). For diarrhoea, we again observed increased risk in the central and parts of the southern region, while decreased risk was observed in the southern region (Figure 3-left panel), and the corresponding posterior probability map highlights areas of excess risk (OR>1). Similarly for pneumonia, the central region displayed increased risk, while the northern region and isolated parts of the southern region depicted lower risk (Figure 4). For all three diseases, areas of excess risk happened to be concentrated in the central region, but overall, the residual spatial association between the three illnesses was weak.

## Discussion

Recent studies have shown significant district-specific spatial variation in childhood fever and diarrhoea in Malawi [11,14], and have attributed this clustering to perennial malaria risk. In addition, others have confirmed a common occurrence of childhood morbidities of fever, diarrhoea and pneumonia and have proposed that malarial infection is a contributing risk factor [1-3,5]. Despite these observations, little research has been carried out to investigate the spatial correlation between the diseases, and the effect of malaria endemicity. In this study, the aim was to investigate these by applying multivariate spatial models to assess subdistrict-specific geographical correlation of childhood fever, diarrhoea and pneumonia, in relation to malaria endemicity.

We observed that the risk of the three illnesses varied with different risk factors, including age of the child, underweight, use of bednet, Vitamin A, and place of residence (urban or rural). The effect of age on the three illnesses is interesting. Generally, all children under the age of 5 years were at increased risk, however, those of an age where they were likely to be weaned (6–23 months) appeared to be at the greatest risk. Very young infants (0–5 months) may have been breastfed, and therefore protected by maternal immunity, and children older than 3 years were less at risk of disease, probably because of acquired immunity. Children who were underweight were at greater risk of diarrhoea. The prevalence of diarrhoea appears to have increased amongst children reported to have had a Vitamin A supplement. This has not been confirmed by other studies and a decrease in morbidity and mortality is the rationale for the provision of Vitamin A supplement. This finding may be a question of reverse causality [20], i.e., children with fever or diarrhoea are more likely to present for health care and thereby receive Vitamin A. This observation, however, warrants further research to see whether treatment seeking behaviour plays an important role.

In this analysis, the risk of all three illnesses, i.e., fever, diarrhoea and pneumonia was found to be associated with malaria endemicity, although this relationship was stronger with fever. It is also evident that malaria endemicity acts as a shared risk factor that increases the risk of other diseases, such as diarrhoea and pneumonia [2,3]. This is especially true at higher levels of endemicity. A recent review [1], indicates that in sub-Saharan Africa where malaria is endemic, the impact of other illnesses including pneumonia and diarrhoea is exacerbated and accordingly co-morbidity of opportunistic diseases is also high [6,7].

The relationship between fever and malaria endemicity is particularly interesting. In malaria-endemic countries, fever is often equated to malarial morbidity for prompt treatment due to non-availability of definitive diagnosis in most places. Although fever as an indicator of malaria infection generates many false positives [21], our findings seem to vindicate the appropriateness of treating children reporting fever with antimalarials [5,22].

As shown in other studies [11], moderate to small subdistrict-specific geographical variation of childhood morbidity of fever, diarrhoea and pneumonia were observed. In the central region the risk patterns were quite similar, indeed, fever and diarrhoea were modestly correlated with high uncertainty and this persisted after adjusting for covariates. This is not surprising as the two are influenced by sanitation-related risk factors [6,11]. Since this correlation persisted after controlling for covariates, the findings suggest common unmeasured covariates influencing both diseases. The correlation between fever and pneumonia, and between diarrhoea and pneumonia was non-significant. Nevertheless, the spatial effects in Figures 2 and 4 do indicate isolated similarities in residual risk between these diseases in some districts. This factor can be explained by severity and/or depth of poverty in these districts, resulting in children exposed to multiple opportunistic illnesses such as diarrhoea and pneumonia [5,7,23]. Overall, the residual spatial associations between the three illnesses was weak because much of the heterogeneity has been explained by covariates included in the model, and to a greater extent the unmeasured or unknown areal covariates may be different for each disease, hence contrasting residual spatial variations.

Excluding malaria endemicity as a risk factor, childhood morbidities are a combined result of several determinants, including the socio-demographic covariates as observed in Table 2. In addition, unobserved covariates as measured by the spatial residuals (Figures 2, 3, 4), may also influence the etiology of childhood morbidities. These could be HIV/AIDS, malnutrition, population density, socio-economic determinants, socio-cultural differences, or indoor air pollution. Kandala *et al. *[11] argued that high population density in the central region affects the child's environment, which in turn influences exposure to diseases, despite overcrowding not being significant in our analysis. Food insecurity associated with drought and flooding in the Shire Valley are among possible explanations for spatial heterogeneity in childhood diseases [14]. Undernutrition, Vitamin A, zinc and other deficiencies also increase susceptibility to common illnesses like malaria, diarrhoea and pneumonia [6]. The residual spatial variation may also represent agricultural and indoor air pollution from biomass combustion, which synergetically contribute to child ailments in particular respiratory illnesses [25]. Further research, though, is definitely needed to verify these assertions. The maps provide a strong foundation for further analysis. The use of interventions such as insecticide treated bednets has a protective effect against malaria, and therefore indirectly for the other two illnesses. Scaling-up of these and related interventions is essential for improved childhood health [26].

Use of DHS data has limitations. Concerns have been raised about potential bias of relying on the ability of mothers to identify and separate symptoms of fever, pneumonia or diarrhoea [21].

Nevertheless, ethnographic studies generally agree that mothers consistently distinguish symptoms of various diseases- the easiest to diagnose being diarrhoea and pneumonia [22]. Secondly, diagnoses of illnesses depended on mother's report (recall) as is common in retrospective surveys. Accuracy and completeness of mother's recall in 19 national DHS surveys found that highly educated women were more accurate in reporting and identification [27]. To provide a consistent sample, recalls were restricted to 14 days prior to the survey. This has been found to provide reliable results in related studies in the country [24]. In addition, the survey was carried out during the summer months (August-October) of 2000. During such time, there is increased likelihood of diarrhoea and malaria, but reduced likelihood of pneumonia. The high prevalence of pneumonia in our results (Table 1), seems to suggest failure in identifying pneumonia which may overlap with malarial fever [5].

HIV is an important confounder in this study and the lack of HIV data in the 2000 survey is a major limitation, thus our results need to be interpreted bearing in mind the co-existence of malaria and HIV. The relationship observed between fever, diarrhoea and pneumonia may be due to the fact that symptoms of HIV include fever, diarrhoea and pneumonia [4]. This is an important research question, which needs thorough investigation when such data become available.

The use of modelled values of malaria prevalence might be prone to errors in the malaria endemicity covariate, resulting in a possible overestimation of standard errors. The use of predicted prevalence values is justified by highly endemic malaria risk in Malawi which has changed little over the years due to lack of sustainable malaria control programmes in the country. As such, modelled estimates provide a realistic pattern of malaria risk, which also agrees with expert opinion [28]. This approach is said to give conservative estimates [29].

In conclusion, the results have depicted similarities in the spatial pattern of fever, diarrhoea and pneumonia. The co-occurrence of fever and diarrhoea was the strongest mainly due to shared risk factor such as malaria endemicity. Although overlaps between fever and pneumonia or between diarrhoea and pneumonia were weak, malaria still played an important role. The magnitude of overlap in symptoms strongly suggest the need to strengthen strategies for the integrated management of childhood illnesses through home and community case management innovations that jointly address such illnesses. Since malaria home and community management are widely accepted in the country, it is possible to include treatments for diarrhoea and pneumonia in the existing malaria dispensing strategies.

## Competing interests

The author(s) declare that they have no competing interests.

## Authors' contributions

LNK conceptualised, collected data, analysed and drafted the manuscript. ASM, CCA and IK participated in the conception and critical review of the manuscript. The authors read and approved the final manuscript.
